# Impact of Clipping versus Coiling on Postoperative Hemodynamics and Pulmonary Edema after Subarachnoid Hemorrhage

**DOI:** 10.1155/2014/807064

**Published:** 2014-04-09

**Authors:** Nobutaka Horie, Mitsutoshi Iwaasa, Eiji Isotani, Shunsuke Ishizaka, Tooru Inoue, Izumi Nagata

**Affiliations:** ^1^Department of Neurosurgery, Nagasaki University School of Medicine, 1-7-1 Sakamoto, Nagasaki 852-8501, Japan; ^2^Department of Neurosurgery, Fukuoka University School of Medicine, 7-45-1 Nanakuma, Jonan-ku, Fukuoka 814-0180, Japan; ^3^Emergency and Critical Care Center, Tokyo Women's Medical University Medical Center East, 2-1-10 Nishi-Ogu, Arakawa-ku, Tokyo 116-8567, Japan

## Abstract

Volume management is critical for assessment of cerebral vasospasm after aneurysmal subarachnoid hemorrhage (SAH). This multicenter prospective cohort study compared the impact of surgical clipping versus endovascular coiling on postoperative hemodynamics and pulmonary edema in patients with SAH. Hemodynamic parameters were measured for 14 days using a transpulmonary thermodilution system. The study included 202 patients, including 160 who underwent clipping and 42 who underwent coiling. There were no differences in global ejection fraction (GEF), cardiac index, systemic vascular resistance index, or global end-diastolic volume index between the clipping and coiling groups in the early period. However, extravascular lung water index (EVLWI) and pulmonary vascular permeability index (PVPI) were significantly higher in the clipping group in the vasospasm period. Postoperative C-reactive protein (CRP) level was higher in the clipping group and was significantly correlated with postoperative brain natriuretic peptide level. Multivariate analysis found that PVPI and GEF were independently associated with high EVLWI in the early period, suggesting cardiogenic edema, and that CRP and PVPI, but not GEF, were independently associated with high EVLWI in the vasospasm period, suggesting noncardiogenic edema. In conclusion, clipping affects postoperative CRP level and may thereby increase noncardiogenic pulmonary edema in the vasospasm period. His trial is registered with University Hospital Medical Information Network UMIN000003794.

## 1. Introduction

Cerebral vasospasm is a leading cause of morbidity and mortality in patients with aneurysmal subarachnoid hemorrhage (SAH) and is potentially treatable. However, no definitive treatment for cerebral vasospasm after SAH has been established, and systemic volume management is still critical for the assessment of vasospasm [1–4]. It is therefore important to develop reliable assessment modalities for close monitoring of the hemodynamic status. As transpulmonary thermodilution (PiCCO Plus; Pulsion Medical Systems, Munich, Germany) can measure important hemodynamic parameters without the need for cardiopulmonary catheterization, it has gained increasing acceptance in many intensive care units [5, 6] and for volume management in patients with SAH [7–12].

Since publication of the findings of the International Subarachnoid Aneurysm Trial, endovascular intra-aneurysmal coil embolization has been used more frequently for the treatment of aneurysmal SAH [13–16]. Endovascular coil embolization is thought to be less invasive than aneurysmal clipping, because the duration of the procedure is shorter and there is no need for craniotomy [17]. However, no previous studies have investigated the impact of treatment strategy (clipping versus coiling) on postoperative hemodynamics and pulmonary edema after SAH. This study aimed to use the data prospectively collected by the SAH PiCCO study group to (1) determine whether there are differences in postoperative hemodynamics and pulmonary edema between patients with SAH who undergo aneurysmal clipping and those who undergo endovascular coiling and (2) investigate the mechanisms underlying postoperative pulmonary edema.

## 2. Methods

### 2.1. Study Population

Patients were included if they had a ruptured cerebral aneurysm diagnosed by cerebral angiography or three-dimensional angiography. The exclusion criteria were (1) <15 years of age, (2) absence of brainstem reflexes, (3) pregnancy, and (4) severe cardiopulmonary dysfunction requiring percutaneous cardiopulmonary support. Patients with rebleeding during the postoperative study period were also excluded because the accuracy of diagnosis of delayed cerebral ischemia (DCI) and the degree of pulmonary edema could be affected by rebleeding. The SAH PiCCO study was a multicenter prospective cohort study of SAH patients admitted to the nine participating Japanese university hospitals. The study was approved by the appropriate ethics committees of all participating institutions, and written informed consent for treatment was obtained from all patients or their next of kin. The study was registered with the University Hospital Medical Information Network (UMIN) Clinical Trials Registry (http://apps.who.int/trialsearch/trial.aspx?trialid=JPRN-UMIN000003794): UMIN-CTR ID UMIN000003794.

All patients admitted to the nine participating institutions with aneurysmal SAH between October 2008 and March 2012 were screened for eligibility. Patients who were monitored using the PiCCO system during the perioperative period were included in the study. All patients underwent aneurysm treatment (clipping or coiling) within 48 hours of the onset of symptoms of SAH. Treatment decisions were at the discretion of the attending physician.

### 2.2. Transpulmonary Thermodilution (PiCCO) Monitoring

All patients were monitored using PiCCO Plus from days 1 to 14 after SAH. A 4-F thermistor-tipped arterial catheter (PV2014L16; Pulsion Medical Systems) was inserted into the femoral or brachial artery. The arterial catheter and a central venous catheter were connected to pressure transducers and to the PiCCO Plus system for monitoring. Global ejection fraction (GEF; normal range 25–35%), cardiac index (CI; 3–5 L/min/m^2^), and systemic vascular resistance index (SVRI; 1700–2400 dyn/sec/m^2^) were measured to assess cardiac contractility and afterload. Global end-diastolic volume index (GEDI; 680–800 mL/m^2^) was measured to assess preload. Extravascular lung water index (EVLWI; 3.0–10.0 mL/kg) and pulmonary vascular permeability index (PVPI; 1.0–3.0) were measured to assess pulmonary parameters. The parameters were determined with continuous cardiac output calibration by triplicate central venous injections of 15 mL of ice-cold saline (<8°C). Cardiac output was calculated by analysis of the thermodilution curve followed by pulse-contour analysis for continuous monitoring. Details of the PiCCO monitoring protocol have been described elsewhere [10, 11, 18].

### 2.3. Postoperative Management

Perioperative care was performed according to the standardized protocol provided by the current American Heart Association guidelines [1]. Patients underwent brain natriuretic peptide (BNP) quantification on postoperative day 1 and laboratory testing of the blood until day 14. Volume management was monitored throughout the analysis until day 14. Intracranial and cerebrospinal fluid pressure were controlled by ventricular, cisternal, or spinal drainage. Blood transfusion was performed when the hemoglobin and hematocrit levels were below the lower limits of the normal ranges (<10 g/dL hemoglobin, <35% hematocrit). Triple-H (hypervolemia, hypertension, and hemodilution) therapy was administered for symptomatic vasospasm at the discretion of the attending physician. DCI was defined as symptomatic cerebral vasospasm or cerebral infarction caused by cerebral vasospasm. In comatose patients, symptomatic vasospasm was defined as cerebral infarction due to vasospasm observed on angiography. Magnetic resonance imaging, computed tomography angiography, and transcranial Doppler ultrasonography were also used to detect vasospasm in patients with DCI.

### 2.4. Statistical Analysis

Data are presented as median values with 95% confidence intervals. Data were tested for normality of distribution and equal standard deviations using GraphPad InStat (Version 3.10; GraphPad Software, USA) to determine whether parametric or nonparametric assumptions should be used for each statistical test. Comparisons between groups were performed using the Mann-Whitney test for continuous variables and the *χ* ^2^ test for categorical variables. Correlations between C-reactive protein (CRP) level and postoperative brain BNP level were analyzed using the Pearson rank correlation test (which measures the linear relationship between two variables), because we expected a linear relationship. Multivariate regression analysis was performed to identify factors associated with high EVLWI (>10 mL/kg) in the early period (days 1–3) and the vasospasm period (days 6–8) using SPSS (Version 15.0; SPSS Japan Inc., Tokyo, Japan). Odds ratios for high EVLWI were adjusted for age, sex, World Federation of Neurological Surgeons (WFNS) grade, Fisher grade, treatment (clipping or coiling), transfusion, triple-H therapy, CRP level, PVPI, GEF, and GEDI. The text and figure legends describe the statistical tests used. Unless stated otherwise, differences were considered statistically significant at *P* < 0.05.

## 3. Results

### 3.1. Patient Characteristics

This study enrolled 204 patients. After exclusion of two patients who underwent surgical trapping with bypass, 202 patients were included in the analyses (Table 1). Clipping was performed in 160 patients and coiling was performed in 42 patients. Surgical clipping was preferably performed for middle cerebral artery aneurysms and endovascular coiling was preferably performed for basilar/vertebral artery aneurysms. There were no significant differences in age, Fisher grade, aneurysm size, preoperative rebleeding rate, DCI, or Glasgow Outcome Scale score between the clipping and coiling groups. However, patients in the coiling group had significantly higher WFNS grades than those in the clipping group (*P* = 0.02). Patients in the clipping group were more likely to receive transfusion (62.5% versus 31.0%; *P* = 0.002) and triple-H therapy (40.0% versus 14.3%; *P* = 0.005) than those in the coiling group.

### 3.2. Postoperative Hemodynamic Parameters after Clipping and Coiling

**Figure 1:**
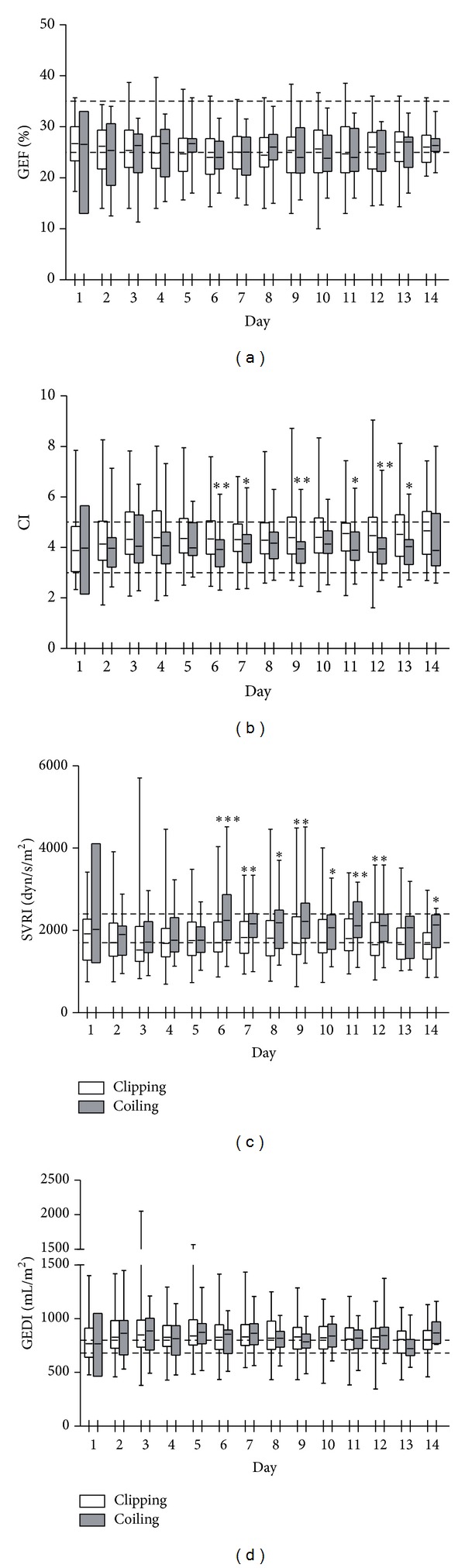
Values for GEF (a), CI (b), SVRI (c), and GEDI (d) for 14 days after SAH. The dotted lines indicate the upper and lower limits of the normal ranges. *P < 0.05, **P < 0.01, ***P < 0.001, Mann-Whitney test. GEF: global ejection fraction; CI: cardiac index; SVRI: systemic vascular resistance index; and GEDI: global end-diastolic volume index.

The time courses of postoperative hemodynamic parameters are shown in Figure 1. GEF, a measure of cardiac contractility, was around the lower limit of the normal range throughout the study period and was not significantly different between the clipping and coiling groups (Figure 1(a)). CI and SVRI were measured to assess afterload (Figures 1(b) and 1(c)). In the early period after SAH, CI was within the normal range and SVRI was around the lower limit of the normal range in both groups. In the vasospasm period (from day 6 onwards), CI was significantly lower and SVRI was significantly higher in the coiling group than in the clipping group. GEDI, a measure of preload, was above the upper limit of the normal range in both groups (Figure 1(d)). These results suggest that afterload mismatch may occur even in the vasospasm period after SAH in cases of aggressive volume loading. In this study, afterload mismatch was more likely to occur in the coiling group than in the clipping group, probably because the coiling group had a higher proportion of patients with poor WFNS grade.

### 3.3. Postoperative Pulmonary Parameters after Clipping and Coiling

**Figure 2:**
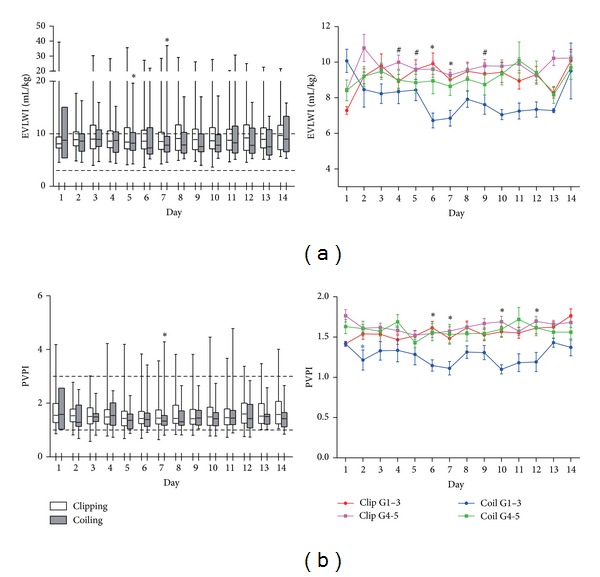
Values for EVLWI (a) and PVPI (b) for 14 days after SAH. The dotted lines indicate the upper and lower limits of the normal ranges. The colored lines show EVLWI and PVPI according to the WFNS grade. *P < 0.05, comparison between the clipping and coiling groups in WFNS grades 1–3; ^#^ *P* < 0.05, comparison between the clipping and coiling groups in WFNS grades 4-5, Mann-Whitney test. EVLWI: extravascular lung water index and PVPI: pulmonary vascular permeability index.

The time courses of postoperative pulmonary parameters are shown in Figure 2. Overall, EVLWI was around the upper limit of the normal range throughout the study period in both groups and was significantly higher in the clipping group than in the coiling group on days 5 and 7 (*P* = 0.049 and *P* = 0.03, resp.; Figure 2(a)). Evaluation of EVLWI according to the WFNS grade showed that EVLWI was significantly higher in the clipping group on days 6 and 7 in patients with WFNS grades 1–3 (*P* = 0.04 and *P* = 0.04, resp.) and on days 4, 5, and 9 in patients with WFNS grades 4-5 (*P* = 0.04, *P* = 0.03, and *P* = 0.03, resp.). However, PVPI was within the normal range throughout the study period in both treatment groups and was significantly higher in the clipping group than in the coiling group on day 7 (*P* = 0.048; Figure 2(b)). Evaluation of PVPI according to the WFNS grade showed that PVPI was significantly higher in the clipping group on days 6, 7, 10, and 12 in patients with WFNS grades 1–3 (*P* = 0.03, *P* = 0.02, *P* = 0.02, and *P* = 0.03, resp.). These results suggest that postoperative pulmonary edema was more likely to occur in the clipping group in the vasospasm period with PVPI elevation.

### 3.4. Associations among CRP Level, Cardiopulmonary Parameters, and Outcome

**Figure 3:**
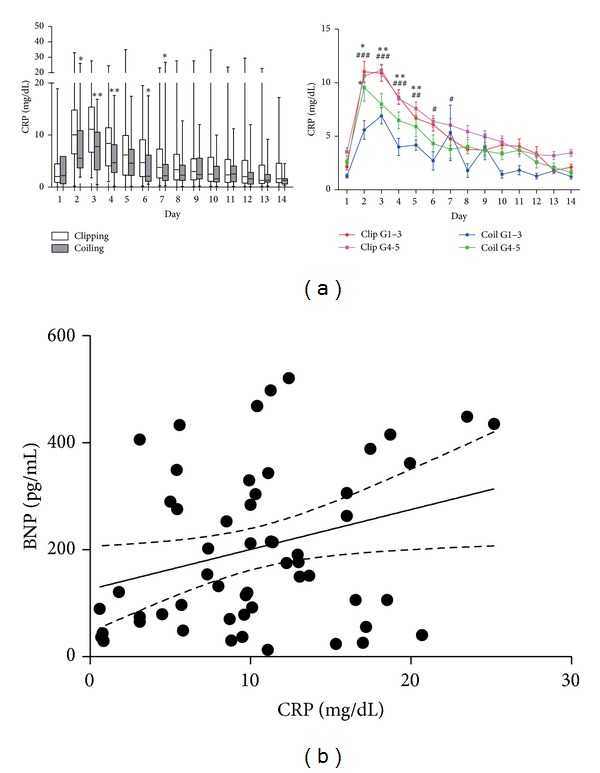
(a) CRP levels for 14 days after SAH. The colored lines show CRP level according to the WFNS grade. *P < 0.05, **P < 0.01, comparison between the clipping and coiling groups in WFNS grades 1–3; ^#^ *P* < 0.05, ^##^ *P* < 0.01, ^###^ *P* < 0.001, comparison between the clipping and coiling groups in WFNS grades 4-5, Mann-Whitney test. (b) Linear regression curves for correlation between the CRP and BNP levels in WFNS grades 4-5. Pearson rank correlation test. CRP: C-reactive protein and BNP: brain natriuretic peptide.

**Figure 4:**
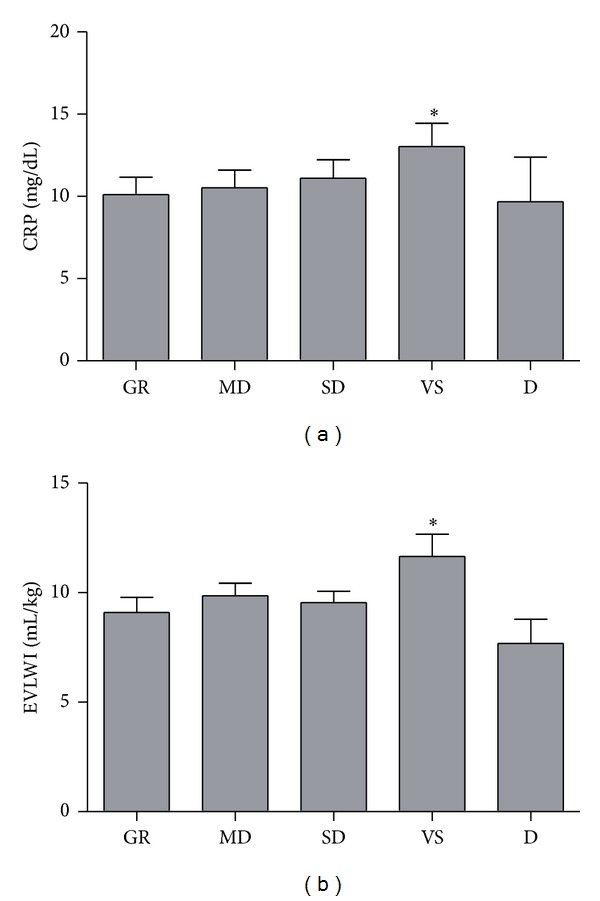
CRP level and EVLWI according to Glasgow Outcome Scale score after SAH. *P < 0.05, one-way analysis of variance, Tukey-Kramer multiple comparisons test. GR: good recovery; MD: moderate disability; SD: severe disability; VS: vegetative state; and D: death.

In both groups, the CRP level increased until day 3 and then decreased gradually (Figure 3(a)). The CRP level was significantly higher in the clipping group than in the coiling group on days 2, 3, 4, 6, and 7 (*P* = 0.03, *P* = 0.006, *P* = 0.005, *P* = 0.02, and *P* = 0.04, resp.). Evaluation of the CRP level according to the WFNS grade showed that there was a moderate-to-weak positive correlation between the CRP level on day 3 and the postoperative BNP level in patients with WFNS grades 4-5 (*r* = 0.29, *P* = 0.03; Figure 3(b)). Evaluation of outcome according to the Glasgow Outcome Scale score at discharge showed that the CRP level on day 3 and EVLWI on day 7 were significantly higher in patients discharged in a vegetative state than in those not discharged in a vegetative state (*P* < 0.05; Figure 4).

Multivariate analysis to identify the risk factors for high EVLWI (>10 mL/kg) showed that PVPI and GEF were independently associated with high EVLWI in the early period, suggesting cardiogenic edema (*P* = 0.001 and *P* = 0.004, resp.; Table 2), and that CRP level and PVPI, but not GEF, were independently associated with high EVLWI in the vasospasm period, suggesting noncardiogenic edema (*P* = 0.022, *P* = 0.001, and *P* = 0.507, resp.; Table 3).

## 4. Discussion

This is the first study to evaluate differences in postoperative hemodynamics and pulmonary edema between patients who underwent surgical clipping and endovascular coiling for aneurysmal SAH. Based on analysis of the PiCCO SAH group database, we present the following findings. First, postoperative hemodynamics are similar in both groups in the early period, and afterload mismatch may occur in the vasospasm period with aggressive volume loading. Previously published reports also suggest that hypervolemia can be harmful in patients with SAH [19, 20]. In this prospective cohort study, patients with severe vasospasm received triple-H therapy combined with precise monitoring of cardiopulmonary function using PiCCO, and prophylactic hypervolemia was induced in selected cases at the discretion of the attending physician. The results of a North American practice survey also showed that physicians were still inducing prophylactic hypervolemia in selected patients with severe vasospasm [21]. The possibility of cardiopulmonary dysfunction due to aggressive hypervolemia should be considered in these patients. Afterload mismatch occurred more frequently in the coiling group, probably because this group had a higher proportion of patients with poor WFNS grade. Second, postoperative pulmonary edema occurred more frequently in the clipping group in the vasospasm period with PVPI elevation. Finally, a high postoperative CRP level may be a risk factor for noncardiogenic pulmonary edema in the vasospasm period.

It is sometimes difficult to evaluate pulmonary edema on X-ray, whereas PiCCO is a useful modality for quantitative evaluation of the status of the lungs [5, 6]. EVLWI was around the upper limit of the normal range throughout the study period, suggesting postoperative accumulation of extravascular lung water even without any abnormal findings on X-ray. Pulmonary edema was detected on X-ray in 19.8% of the patients in this study. The true incidence of pulmonary complications after SAH remains unclear because of the lack of quantitative hemodynamic measurements to date.

The International Subarachnoid Aneurysm Trial reported lower rates of death and disability after endovascular treatment of ruptured intracranial aneurysms than after neurosurgical treatment [13–16]. Based on these results, the treatment of patients with ruptured intracranial aneurysms has changed significantly over recent years. In many centers, coiling has become the treatment modality of choice when both coiling and clipping are considered suitable in a particular patient. However, very few reports have shown differences between clipping and coiling in terms of invasiveness [17, 22]. As the brain is not manipulated during endovascular coiling, the risk of brain damage may be reduced compared with clipping. Moreover, the time of operation under anesthesia is much shorter for coiling than for clipping. These differences could affect the catecholamine surge during the acute stage after SAH, thereby affecting postoperative cardiopulmonary function [23, 24], which is very important for the volume management of vasospasm after SAH.

Neurogenic pulmonary edema is a clinical syndrome characterized by acute onset of pulmonary edema following a significant central nervous system insult such as SAH, spinal cord injury, traumatic brain injury, or intracranial hemorrhage [25]. Neurogenic pulmonary edema is thought to be caused by a catecholamine surge that results in cardiopulmonary dysfunction [26], and several mechanisms have been proposed: neurocardiac [27], neurohemodynamic [28], blast theory [29], and pulmonary venule adrenergic hypersensitivity [30]. Our data show that EVLWI was initially high in both groups, probably because of a catecholamine surge after the onset of SAH. However, the time course of EVLWI was different between patients who underwent clipping and coiling. EVLWI stayed high in the clipping group but gradually improved in the coiling group. In this study, we focused on CRP level because the postoperative CRP level was significantly higher in the clipping group than in the coiling group. Recently, a high postoperative CRP level has been reported to be a predictive factor for poor outcome after SAH, although the mechanism underlying this association is still undetermined [31, 32]. In this study, we found two types of pulmonary edema after SAH: cardiogenic pulmonary edema due to poor cardiac contractility in the early period and noncardiac pulmonary edema due to elevation of the postoperative CRP level, which mainly occurred after clipping. This is consistent with previously reported findings [10]. The significant correlation between the CRP level (which increases for 24–48 hours after an inflammatory insult) and the postoperative BNP level strongly supports our hypothesis that the postoperative CRP level affects cardiopulmonary function after SAH. Although there is no established evidence of associations among CRP level, EVLWI, and BNP, we consider that systemic inflammatory response syndrome in response to the brain injury after SAH [33–35] may contribute to the cardiopulmonary complications. Further hemodynamic data are needed to clarify the underlying mechanisms.

BNP is a hormone primarily produced in the left cardiac ventricle in response to cardiac wall stretch, which causes diuresis through direct natriuretic action, increased cardiac output, or decreased aldosterone level [36]. In a small series of SAH patients, plasma concentrations of BNP were found to be elevated [37–39], but the reasons for this are still unclear. Our findings indicate that the increased postoperative CRP level could result in increased elevation of the BNP level, via increased EVLWI due to cardiogenic or noncardiogenic pulmonary edema. Takotsubo cardiomyopathy, which is associated with the catecholamine surge after SAH [40], may also play a role in the cardiac insufficiency. Finally, we found that the postoperative CRP level in the early period and EVLWI in the vasospasm period were significantly higher in patients with a vegetative state after SAH than in those not in a vegetative state. This indicates that postoperative CRP level can predict pulmonary complications that affect morbidity after SAH.

Taken together, careful monitoring of the hemodynamic and pulmonary parameters until the end of the vasospasm period is mandatory to avoid development of cardiac insufficiency or pulmonary complications, which could contribute to the outcome after SAH.

Several limitations of this study should be discussed. First, catecholamine levels were not collected in the PiCCO study. The BNP level was recorded once, within 3 days after SAH (postoperative day 1), and the time course of the BNP level was therefore not available. Second, cardiac function was evaluated using only PiCCO data, and regular echocardiography or electrocardiography evaluations were not performed. Nevertheless, bedside monitoring using PiCCO is a powerful, established tool for the assessment of cardiopulmonary hemodynamics [5–11]. Third, distribution of the aneurysm location was different between patients who undergo clipping and coiling, which could affect cardiopulmonary function [41, 42]. Impact of aneurysm location on cardiopulmonary dysfunction could be clarified by another substudy. Fourth, sedation and mechanical ventilation data were not recorded in the PiCCO study, and parameters affected by mechanical ventilation were therefore not analyzed in this study. It has previously been reported that EVLWI is not affected by mechanical ventilation [43]. Finally, intracranial pressure monitoring was not performed or recorded in this study which might affect EVLWI [41].

In conclusion, this is the first study to evaluate the differences in postoperative hemodynamics and pulmonary parameters between clipping and coiling for aneurysmal SAH. Postoperative hemodynamics were similar in both groups in the early period, and afterload mismatch sometimes occurred in the vasospasm period. Clipping may increase noncardiogenic pulmonary edema in the vasospasm period, resulting from the increased postoperative CRP level. The postoperative CRP level may predict pulmonary complications after SAH.

## Figures and Tables

**Table 1:** Clinical characteristics of patients.

	Clipping (%)	Coiling (%)	*P* value
*N*	160	42	
Age	63.4 ± 13.1	61.9 ± 11.8	n.s
Male sex	49(30.6)	16(38.1)	n.s
WFNS grade			0.02
I	17(10.6)	3(7.1)	
II	30(18.8)	5(11.9)	
III	11(6.9)	1(2.4)	
IV	43(26.9)	8(19.0)	
V	59(36.9)	25(59.5)	
Fisher grade			n.s
1	1(0.6)	0(0.0)	
2	9(5.6)	2(4.8)	
3	92(57.5)	26(61.9)	
4	58(36.3)	14(33.3)	
Aneurysm size	6.6 ± 5.5	7.1 ± 4.1	n.s
Aneurysm location			
ACA	47(29.4)	11(26.2)	n.s
MCA	61(38.1)	0(0)	<0.0001
ICA	46(28.8)	9(21.4)	n.s
BA/VA	6(3.4)	22(52.4)	<0.0001
Rebleeding	3(1.8)	1(2.3)	n.s
Transfusion	100(62.5)	13(31.0)	0.002
Triple-H therapy	64(40.0)	6(14.3)	0.005
DCI	36(22.5)	10(23.8)	n.s
GOS			n.s
GR	38(23.8)	8(19.0)	
MD	43(26.9)	8(19.0)	
SD	45(28.1)	14(33.3)	
VS	25(15.0)	9(21.4)	
D	9(5.6)	3(7.1)	

WFNS: World Federation of Neurological Surgeons; DCI: delayed cerebral ischemia; GOS: Glasgow Outcome Scale; GR: good recovery; MD: moderate disability; SD: severe disability; VS: vegetative state; D: death; ACA: anterior communicating artery; MCA: middle cerebral artery; ICA: internal carotid artery; and BA/VA: basilar artery/vertebral artery; n.s: not significant.

**Table 2:** ORs for high EVLWI (>10 mL/kg) in the early period.

Variable	Adjusted OR	95% CI	*P* value
Age	0.99	0.94–1.05	0.842
Sex female	2.85	0.43–19.10	0.280
WFNS grade	1.27	0.68–2.38	0.457
CT Fisher grade	0.56	0.19–1.64	0.292
Clipping	1.31	0.29–5.84	0.726
Transfusion	1.95	0.28–13.53	0.500
CRP day 3	0.97	0.85–1.12	0.687
PVPI day 3	27.54	3.59–211.42	0.001
GEF day 3	0.75	0.62–0.91	0.004

WFNS: World Federation of Neurological Surgeons; CRP: C-reactive protein; PVPI: pulmonary vascular permeability index; and GEF: global ejection fraction.

**Table 3:** ORs for high EVLWI (>10 mL/kg) in the vasospasm period.

Variable	Adjusted OR	95% CI	*P* value
Age	1.04	0.99–1.09	0.131
Sex female	2.77	0.57–13.4	0.206
WFNS grade	1.15	0.67–1.98	0.614
CT Fisher grade	0.37	0.12–1.22	0.103
Clipping	1.19	0.26–5.54	0.821
Transfusion	1.53	0.29–8.13	0.621
Triple H	0.80	0.23–2.83	0.729
CRP day 3	1.15	1.02–1.29	0.022
PVPI day 7	35.97	4.27–303	0.001
GEF day 7	1.05	0.91–1.22	0.507

WFNS: World Federation of Neurological Surgeons; CRP: C-reactive protein; PVPI: pulmonary vascular permeability index; and GEF: global ejection fraction.

## Abbreviations

SAH: Subarachnoid hemorrhage
DCI: Delayed cerebral ischemia
GEF: Global ejection fraction
CI: Cardiac index
SVRI: Systemic vascular resistance index
GEDI: Global end-diastolic volume index
EVLWI: Extravascular lung water index
PVPI: Pulmonary vascular permeability index
CRP: C-reactive protein
BNP: Brain natriuretic peptide
WFSN: World Federation of Neurological Surgeons.
